# Simultaneous Heterotrophic Nitrification and Aerobic Denitrification by *Chryseobacterium* sp. R31 Isolated from Abattoir Wastewater

**DOI:** 10.1155/2014/436056

**Published:** 2014-06-02

**Authors:** Pradyut Kundu, Arnab Pramanik, Arpita Dasgupta, Somnath Mukherjee, Joydeep Mukherjee

**Affiliations:** ^1^Department of Civil Engineering, Jadavpur University, Kolkata 700 032, India; ^2^School of Environmental Studies, Jadavpur University, Kolkata 700 032, India

## Abstract

A heterotrophic carbon utilizing microbe (R31) capable of simultaneous nitrification and denitrification (SND) was isolated from wastewater of an Indian slaughterhouse. From an initial COD value of 583.0 mg/L, 95.54% was removed whilst, from a starting NH_4_^+^-N concentration of 55.7 mg/L, 95.87% was removed after 48 h contact. The concentrations of the intermediates hydroxylamine, nitrite, and nitrate were low, thus ensuring nitrogen removal. Aerobic denitrification occurring during ammonium removal by R31 was confirmed by utilization of both nitrate and nitrite as nitrogen substrates. Glucose and succinate were superior while acetate and citrate were poor substrates for nitrogen removal. Molecular phylogenetic identification, supported by chemotaxonomic and physiological properties, assigned R31 as a close relative of *Chryseobacterium haifense*. The NH_4_^+^-N utilization rate and growth of strain R31 were found to be higher at C/N = 10 in comparison to those achieved with C/N ratios of 5 and 20. Monod kinetic coefficients, half saturation concentration (*K*_*s*_), maximum rate of substrate utilization (*k*), yield coefficient, (*Y*) and endogenous decay coefficient (*K*_*d*_) indicated potential application of R31 in large-scale SND process. This is the first report on concomitant carbon oxidation, nitrification, and denitrification in the genus *Chryseobacterium* and the associated kinetic coefficients.

## 1. Introduction

Conventionally, ammonium from abattoir wastewater is removed in sequential operations through alternate aerobic and anoxic periods over time [1–3]. Majority of the carbon is degraded during the aerobic nitrification phase and the residual carbon is used up during the anoxic denitrification phase [4]. However, such systems are prone to operational hindrances due to reduced rate of nitrification and the difficulty to separate nitrification and denitrification reaction processes. Meyer et al. [5] noted that nitrification and denitrification would be incompletely coupled to each other due to nonformation of aerobic/anoxic zones within the microbial aggregates, resulting in buildup of NO_*x*_^−^ in the reactor. In comparison with the conventional nitrification and denitrification of abattoir wastewater, several advantages of a shortcut nitrogen removal method via nitrite, through partial nitrification followed by denitrification (PND), have been reported recently [6].

Zheng et al. [7] observed that both nitrification and denitrification could take place concomitantly in a single reactor under identical reaction conditions through a process known as simultaneous nitrification and denitrification (SND). This procedure neither requires large reactor volume nor incurs high energy costs necessary for circulating liquid between aerobic and anoxic systems. Furthermore, SND processes are characterized by increased nitrogen removal rates and decreased reaction time. The key point in the PND process is maintaining the production rate of nitrite by ammonium oxidizing bacteria (AOB) higher than the production rate of nitrate by nitrite oxidizing bacteria (NOB) so that nitrite accumulation can be achieved [6]. In contrast, maintenance of a population balance is not necessary in SND as the two reactions (nitrification and denitrification) proceed under identical reaction conditions. Thus, SND is advantageous over PND. Furthermore, the SND process prevents accumulation of NO_*x*_^−^ in the system [8]. Discovery of several bacteria capable of carrying out SND has generated considerable scientific interest in developing efficient biological nitrogen removal process in a single reaction vessel [7]. Examples of such organisms isolated from different sources include* Agrobacterium* sp. [9],* Rhodococcus* sp. [10],* Alcaligenes faecalis* [11],* Diaphorobacter* sp. [12],* Bacillus subtilis* [13],* Bacillus methylotrophicus* [14],* Acinetobacter calcoaceticus* [15],* Marinobacter* sp. [7], and* Klebsiella pneumoniae* [16]. Careful review of the literature shows that any bacterium capable of performing SND has not been isolated from slaughterhouse wastewater. It is further observed that there are gaps in understanding the role of microorganisms in the nitrogen removal processes occurring in abattoir wastewater. For example, Yilmaz et al. [8] reported that phosphate accumulating organisms (mainly* Accumulibacter* spp.) occurring in abattoir wastewater were responsible for the denitrification. However, microorganisms that reduced nitrate to nitrite, before* Accumulibacter* spp. could carry out denitrification from nitrite to nitrogen gas, could not be identified by the authors.

Thus, investigations on isolation of nitrifying and denitrifying bacteria from slaughterhouse wastewater, establishing their taxonomical identity, characterizing their metabolic patterns, and identifying their potential application in large-scale nitrogen removal processes are necessary. In our previous study [17] we isolated a microbe from slaughterhouse wastewater and identified it as* Achromobacter xylosoxidans*. This bacterium, previously known as a clinical isolate was, for the first time, demonstrated to have nitrification activity. Interestingly, anaerobic denitrification, a property which is regarded as an intrinsic characteristic of the* Achromobacter* genus, was conserved. In continual pursuance of our search for novel nitrifiers/denitrifiers, the present paper describes the isolation of a new nitrifying-denitrifying bacterium capable of performing SND from wastewater emanating from a small-size rural slaughterhouse in India. This communication further reports its taxonomical identification, nitrogen removal pathway characterization, and kinetics of substrate utilization and growth.

## 2. Materials and Methods

### 2.1. Seed Acclimatization

Sludge collected from the pond discharging wastes of M/s Mokami Slaughterhouse situated in Nazira, South 24 Parganas, West Bengal, India, was acclimatized to the laboratory conditions by cultivation in an aerated vessel for three months as described [17].

### 2.2. Isolation of the Bacterium and Development of Pure Culture

Ten predominant nitrifying bacteria were isolated from the seed acclimatization vessel as described [17]. Nitrification activities and preliminary taxonomic characterization of the isolates are presented as Supplementary File 1 in Supplementary Material available online at http://dx.doi.org/10.1155/2014/436056. Amongst them, the bacterium showing maximum nitrification activity (strain R31) was selected for further studies.

### 2.3. Molecular Phylogenetic Analysis of Strain R31

Extraction of DNA, amplification by polymerase chain reaction (PCR), and sequencing of the 16S rRNA gene were outsourced to Scigenome Labs, Kochi, India. DNA was extracted from the liquid culture of R31 grown in Luria broth following Sambrook et al. [19]. Amplification of the 16S rRNA gene of strain R31 was done with forward primer 27F (5′-AGA GTT TGA TCM TGG CTC AG-3′) and reverse primer 1492R (5′-GGT TAC CTT GTT ACG ACT T-3′) [20]. PCR was performed in a 50 *μ*L reaction mixture containing 5 *μ*L of 10x PCR buffer with 1.5 mM MgCl_2_, 4 *μ*L of 10 mM dNTP, 50 pmol of each oligonucleotide primers, and 1 *μ*L of 3U Taq DNA polymerase. The conditions of PCR were initial denaturation of 5 min at 94°C, subsequently 30 incubation cycles each comprising 30 seconds denaturation at 94°C, 45 sec annealing at 57°C, 1.5 min elongation at 72°C, and a final 7 min elongation at 72°C. The amplicon was electrophoresed in a 1% agarose gel and visualized under UV light. Next, the amplicon was purified using Nucleospin purification column (Macherey-Nagel, Germany). The amplicon was sequenced using an Applied Biosystems (USA) ABi 3730XL Genetic Analyzer. The PCR primers used for amplification were applied for sequencing the amplicon. Phylogenetic analysis of the 16S rRNA gene sequence was done according to Kundu et al. [17].

### 2.4. Fatty Acid Methyl Ester (FAME) Analysis of Strain R31

FAME analysis of the isolate was outsourced to the Microbial Type Culture Collection and Gene Bank (MTCC), Institute of Microbial Technology, Chandigarh, India. Analysis was done using standard gas chromatography and applying the Sherlock MIS system.

### 2.5. Determination of Physiological Characteristics of Strain R31

Physiological characteristics of the strain were determined following the Bergey's manual [21]. Presence of flagella, anaerobic growth, sodium chloride tolerance, aesculin hydrolysis, indole production, catalase activity, gelatin liquefaction, Tween 80 hydrolysis, Simmons citrate utilization, deoxyribonuclease activity, Voges-Proskauer test, methyl red test, starch hydrolysis, *β*-galactosidase activity, urease test, and tyrosine hydrolysis were carried out following the methods described in Kundu et al. [17]. Nitrate reduction test was performed to determine whether the isolate (R31) elaborated the enzymes nitrate reductase and nitrite reductase. These two enzymes catalyze two reactions required for converting nitrate to the end product, nitrogen gas. Aliquot from a pure culture of the isolate R31 was aseptically inoculated to a sterile tube containing nitrate broth with an inverted Durham's tube. The inoculated tube was incubated at 37°C for 24 hours. Finally the reductions of nitrate and nitrite were ascertained by addition of naphthylamine and sulphanilic acid solutions to the nitrate broth followed by adding a small amount of zinc dust [22]. Acid production from various carbohydrates was done following Hinz et al. [23] and utilization of sole carbon, nitrogen, and amino acids was done according to Gutnick et al. [24] while growth in various media, different temperatures, and pH as well as testing of antibiotic resistance and susceptibility were done in accordance with standard procedures as follows. To determine the optimum temperature and pH for growth, the isolate was cultured in Luria-Bertani medium and incubated at 37°C with shaking (100 rpm) for 48 hours. Bacterial growth was confirmed by measuring the optical density of the culture at 600 nm. Susceptibility to antibiotics was determined by the routine agar-diffusion plate technique using disks impregnated with following antibiotics: vancomycin (30 *μ*g), lincomycin (15 *μ*g), kanamycin (30 *μ*g), neomycin (30 *μ*g), oleandomycin (15 *μ*g), ampicillin (10 *μ*g), benzylpenicillin (10 units), streptomycin (10 *μ*g), polymyxin B (300 units), gentamicin (50 *μ*g), tetracycline (30 *μ*g), carbenicillin (100 *μ*g), and chloramphenicol (30 *μ*g). Lysine and arginine decarboxylase activities were measured following Møller [25], whilst production of flexirubin pigment was tested according to Fautz and Reichenbach [26]. Hydrogen sulphide production was detected by visualizing the blackening of SIM medium [27]. All tests were done thrice in triplicate sets.

### 2.6. Carbon Oxidation and Ammonium Stabilization by Strain R31

Carbon oxidation and ammonium stabilization were assessed in batch cultures by cultivating strain R31 in a basal medium (all units g/L): K_2_HPO_4_ 14; KH_2_PO_4_ 6; MgSO_4_·7H_2_O 0.2; trace mineral solution 2 mL; pH (at 25°C) = 7 ± 0.2. The trace mineral solution consisted of (all units g/L), EDTA 0.01; ZnSO_4_·7H_2_O 0.0001; CaCl_2_·2H_2_O 0.1; MnCl_2_·2H_2_O 0.008; FeCl_3_·6H_2_O 0.71; (NH_4_)_6_Mo_7_O_24_ 0.00011; CuSO_4_·5H_2_O 0.0001; CoCl_2_·6H_2_O 0.2. The carbon (glucose) concentration was 1.251 g/L (carbon substrate 500 mg/L) while nitrogen (ammonium chloride) was 0.191 g/L (nitrogen substrate 50 mg/L) forming C/N ratio (w/w) of 10. The sterilized media were inoculated with 1% (v/v) of the isolate and incubated at 37°C with shaking (100 rpm) for 48 h. Aliquots were removed from each Erlenmeyer flask at four hour intervals and growth was recorded by measuring OD_600_. Next, the liquid was centrifuged at 10,000 rpm for 10 minutes and the supernatant was analyzed for COD (by closed reflux method using dichromate), ammonia, nitrite, and nitrate nitrogen by using an expandable ion electrode analyzer (Orion EA 940, Thermo Fisher Scientific, USA). Medium pH was measured using Cyberscan 510 pH meter (Eutech Instruments, Singapore). Hydroxylamine was measured spectrophotometrically according to Frear and Burrell [28]. Experiments were done thrice in triplicate sets.

### 2.7. Influence of Carbon and Nitrogen Substrates on Nitrogen Removal by Strain R31

Effect of different types of carbon and nitrogen substrates and C/N ratios on nitrogen removal is an important consideration in wastewater treatment. So, the influence of the nature of substrates and their relative concentrations on nitrogen removal ability of isolate R31 was investigated through batch experiments.

#### 2.7.1. Carbon Substrates

From a survey of literature [7, 9, 11–15, 29–33] on the use of various carbon sources for bacteria carrying out SND, the most common substrates applied by other investigators appeared to be glucose, sodium succinate, sodium acetate, and sodium citrate. The influence of various carbon substrates was considered at constant ammonia nitrogen concentration of 50 mg/L and C/N ratio of 10.0. Glucose (1.251 g/L), sodium succinate (1.688 g/L), sodium acetate (1.709 g/L), and trisodium citrate (1.792 g/L) were used as different carbon substrates in the basal medium described in Section 2.6 taking into account the fixed ammonia nitrogen concentration and the corresponding constant C/N ratio.

#### 2.7.2. Nitrogen Substrates

Sodium nitrite (0.303 g/L) and sodium nitrate (0.303 g/L) were used in place of ammonium chloride in the basal medium described in Section 2.6 to ascertain the denitrification pathway of R31. The carbon substrate concentration and C/N ratio were fixed at 500 mg/L and 10.0, respectively.

#### 2.7.3. C/N Ratios

The influence of various C/N ratios, namely, 5, 10, and 20, on heterotrophic nitrification-aerobic denitrification by strain R31 was considered at constant ammonium nitrogen concentration of 50 mg/L. The C/N ratio was fixed by adjusting the amount of the carbon substrate. Thus, the amounts of glucose used were 0.625 g/L (C/N = 5), 1.251 g/L (C/N = 10), and 2.502 g/L (C/N = 20). Each flask containing sterilized media was inoculated with the isolate (1% v/v) and incubated at 37°C with shaking (100 rpm) for 48 h. Aliquots were removed from each Erlenmeyer flask at four hour intervals and growth was recorded by measuring OD_600_. The liquid was centrifuged at 10,000 rpm for 10 minutes and analyzed for ammonia nitrogen. All experiments were done thrice in triplicate sets.

### 2.8. Evaluation of Kinetic Parameters

To study simultaneous carbon oxidation, nitrification, and denitrification kinetics, experiments were carried out in four different sets; wherein each set consisted of twelve Erlenmeyer flasks. The C/N ratio in each flask was maintained at 10.0 with appropriate amounts of glucose (carbon substrate) and ammonium chloride (nitrogen substrate). All flasks containing sterilized basal media were inoculated with the isolate (1% v/v) and incubated at 37°C with shaking (100 rpm) for 48 h. One flask from each set was removed from the shaker every four hours. The culture was centrifuged at 10,000 rpm for 10 minutes and the supernatant was analyzed for COD, ammonia nitrogen, nitrate nitrogen, pH, and MLVSS (mixed liquor volatile suspended solids), which was measured gravimetrically in a muffle furnace at 550 ± 50°C). Experiments were done thrice.

#### 2.8.1. Kinetics of Substrate Removal and Growth

Estimation of the kinetic constants for carbon and ammonia utilization, such as half saturation concentration (*K*_*s*_) and maximum substrate utilization rate (*k*), as well as determination of the growth kinetic constants such as yield coefficient (*Y*) and the endogenous decay coefficient (*K*_*d*_) were done by applying Lawrence and McCarty's modified Monod equation [34]. The results so obtained were fitted in to a straight line in accordance with the Lineweaver-Burk plot for determination of the kinetic constants.

## 3. Results and Discussion

### 3.1. Basic Taxonomical Identification of Strain R31

**Figure 1:**
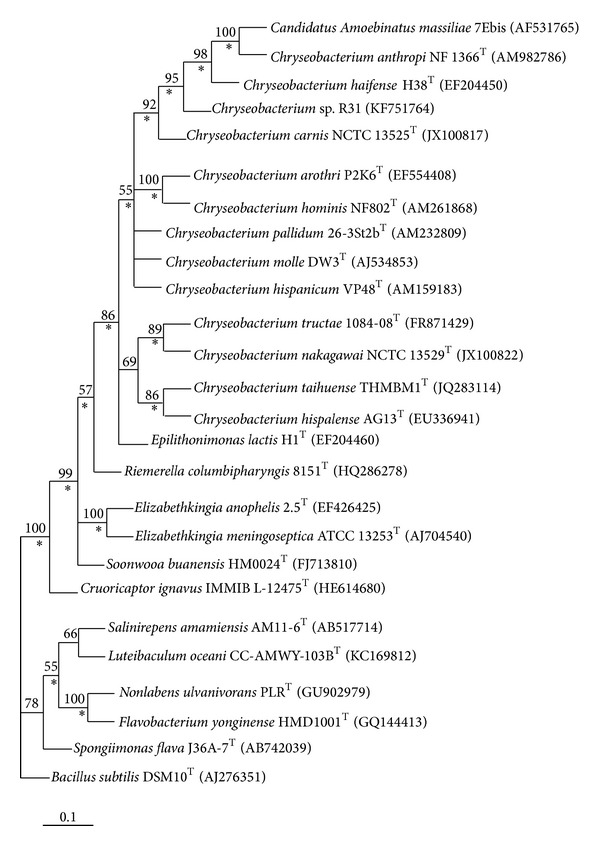
Unrooted phylogenetic tree obtained by the neighbor-joining (NJ) method based on 16S rRNA gene sequences depicting the position of strain* Chryseobacterium *sp. R31 amongst its phylogenetic neighbors. Numbers at nodes designate levels of bootstrap support (%) based on a NJ analysis of 1000 resampled datasets; only values higher than 50% are displayed. Asterisks denote branches that were obtained using the maximum parsimony and maximum likelihood algorithms. NCBI accession numbers are provided in parentheses. Bar = 0.1 nucleotide substitutions per site. The sequence of* Bacillus subtilis* DSM 10 (AJ276351) was applied as an outgroup.

The physiological characteristics of isolate R31 are listed in Supplementary File 2. Small, circular colonies with entire edges were observed when R31 was grown on nutrient agar plates. The colonies were glossy, raised, and yellowish-golden in color. On the basis of nucleotide homology and phylogenetic analysis, the isolate was shown to be significantly similar to* Chryseobacterium haifense*. Identity analysis on the EZ taxon server [35] revealed that the 16S rRNA gene sequence had the closest similarity (98.25%) to the gene sequence of the type strain of* Chryseobacterium haifense* (Supplementary File 3). The phylogenetic position of the isolate (R31) having NCBI Genbank accession number KF751764 is shown in the dendogram (Figure 1). Strain R31 emerged as a distinctive phylogenetic line from the cluster containing the type of strains of* Chryseobacterium *species shown in Supplementary File 3. This differentiation was corroborated by considerable branch length and a high bootstrap value (95%). The phylogenetic position of strain R31 was further supported by the maximum parsimony (MP) and maximum likelihood (ML) analyses. An analogous delineation of the strain was found applying the MP and ML algorithms, which was again confirmed by high bootstrap values (70% for MP and 98% for ML) and significant branch lengths. The result of the fatty acid methyl ester (FAME) analysis is shown in Supplementary File 4. The predominant cellular fatty acids of isolate R31 were 15:0 iso (40.40%), 15:0 anteiso (19.44%), and 17:0 iso 3-OH (11.13%) which closely matched those of* Chryseobacterium haifense* ^*T*^ *: *15:0 iso (41.6%), 15:0 anteiso (16.6%), and 17:0 iso 3-OH (10.3%) [36]. In the nitrate reduction test, gas formation was observed in Durham's tube indicating that the isolate R31 had the ability to convert nitrate to nitrite and then to nitrogen gas. The isolate (R31) was found positive for both nitrate reductase and nitrite reductase. Similar conclusions were drawn by Fernández et al. [37].

A number of physiological characteristics corresponded with the standard species description of* C. haifense* such as having Gram-negative reaction, being nonmotile without any flagella, being obligately aerobic, having temperature ranges of growth, having growth at 4°C, having NaCl requirements for growth, being oxidase positive, being urease negative, being lysine decarboxylase negative, being aesculin and gelatin hydrolysis positive, and being flexirubin production negative. On the other hand the physiological traits of strain R31 that did not agree the standard species description of* C. haifense* were having acid production from carbohydrates, being catalase negative, being nitrate reduction positive, being *β*-galactosidase negative, being indole production negative, and being H_2_S production positive. Strain R31 may be a new member of the* Chryseobacterium* genus and further confirmation can be made through DNA-DNA hybridization tests.

### 3.2. Simultaneous COD and Nitrogen Removal by Strain R31

#### 3.2.1. Carbon Oxidation and Ammonium Stabilization by Strain R31

**Figure 2:**
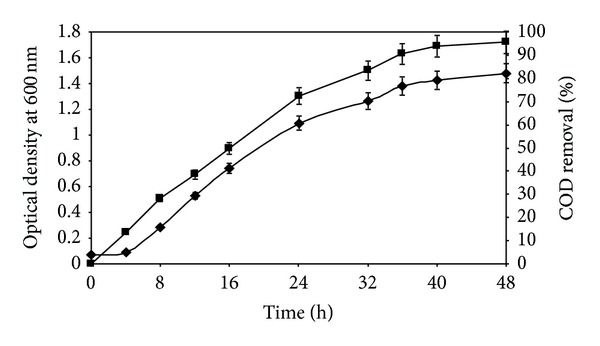
Time profile of carbon oxidation and growth of* Chryseobacterium *sp. R31. Growth* (filled diamonds)* and COD removal (%)* (filled squares)*. Error bars represent one SD (*n* = 9).

Figure 2 shows the profile of COD removal with respect to the time of contact. After 48 h, 95.54% of COD was removed from the initial concentration of 583.0 mg/L, indicating heterotrophic nutritional characteristic of isolate R31. Stabilization of the utilizable component of the basal medium within the reaction period was indicated by the asymptotic nature of the curve after 48 h. Growth kinetics corresponding to carbon oxidation is also shown in Figure 2. The lag phase lasted for approximately 4 h following which isolate R31 grew exponentially for 30 h. Highest biomass production was observed around 48 h following which the microorganism attained the stationary phase of growth. Yang et al. [13] demonstrated growth of heterotrophic nitrifying-denitrifying* Bacillus subtilis* A1 and the nitrogen removal process consumed most of the organic substrates in the media, yielding COD removal efficiencies of 71.0 ± 0.5%, 67.1 ± 0%, 64.5 ± 1.5%, and 63.9 ± 1.8% for acetate, glucose, citrate, and succinate, respectively. Khardenavis et al. [12] attained COD removal of 85–93% using* Diaphorobacter* sp. These values were lower than that obtained in this study.

**Figure 3:**
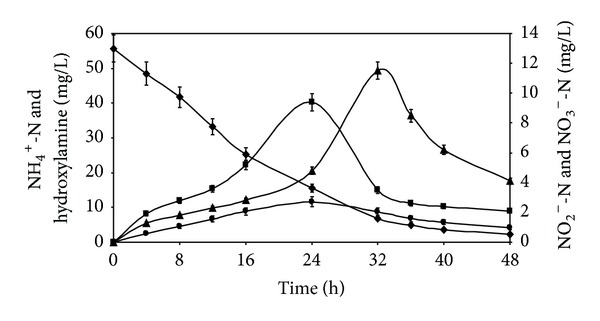
Time profile of ammonium oxidation by* Chryseobacterium *sp. R31. Ammonium nitrogen* (filled diamonds)*, nitrite nitrogen* (filled squares)*, nitrate nitrogen* (filled triangles)*, and hydroxylamine* (filled circle)*. Error bars represent one SD (*n* = 9).

The profile of ammoniacal nitrogen utilization by isolate R31 with relation to time is also shown in Figure 3. After a contact period of 48 h, 95.87% of NH_4_^+^-N was consumed demonstrating the heterotrophic nitrification ability of the isolate. Figure 3 further depicts that the initial ammonia utilization rate was high and maximum stabilization of ammonium in solution was attained within 32 h, after which the utilization declined and ammonium was removed marginally. Exhaustion of ammonium led to reduced availability of the nitrogen substrate to the microorganism. Figure 3 also represents the corresponding nitrite nitrogen (NO_2_^−^-N) and nitrate nitrogen (NO_3_^−^-N) concentrations with respect to time. This figure shows that ammonium was substantially converted to nitrite and, after a reaction period of 24 h, the nitrite level in the reactor was maximum corresponding to 71.99% ammonium utilization. Nitrate concentration initially increased with time confirming heterotrophic nitrification by strain R31. Nitrate concentration reached its peak value at 32 h when 87.79% of ammonium was consumed. The decrease in nitrate concentration after reaching a peak value ascertained the denitrification process. Nitrate was utilized by the isolate after ammonium was exhausted. Hydroxylamine was detected in reduced amounts possibly due to the unstable nature of hydroxylamine and rapid transformation to the next intermediate, nitrite [14]. Simultaneous utilization of organic matter and ammonia degradation indicated R31 to be competent of heterotrophic nitrification as well as aerobic denitrification. Hydroxylamine, nitrite, and nitrate accumulation occurred with the decrease of ammonia nitrogen. The concentrations of the three intermediates were low, thus ensuring nitrogen removal. The profile of hydroxylamine, nitrite, and nitrate formation was similar to that observed during SND processes occurring in* Agrobacterium* sp. [9] as well as* Klebsiella pneumonia* [16]. The amount of ammonium removed (95.87% in 48 h) was higher than that striped off by* Bacillus subtilis* (58.4 ± 4.3% in 60 h) [13],* Bacillus methylotrophicus* (51.03% in 54 h) [14], and* Marinobacter* sp. F6 (48.62%) [7]. The ammonium utilization rate (1.15 mg/L/h) was similar to that obtained for* Pseudomonas alcaligenes *sp. AS-1 (1.15 mg/L/h) [10] and higher than the corresponding value of* Bacillus* sp. LY (0.43 mg/L/h) [10].

#### 3.2.2. Influence of Carbon Substrates on Nitrogen Removal by Strain R31

**Figure 4:**
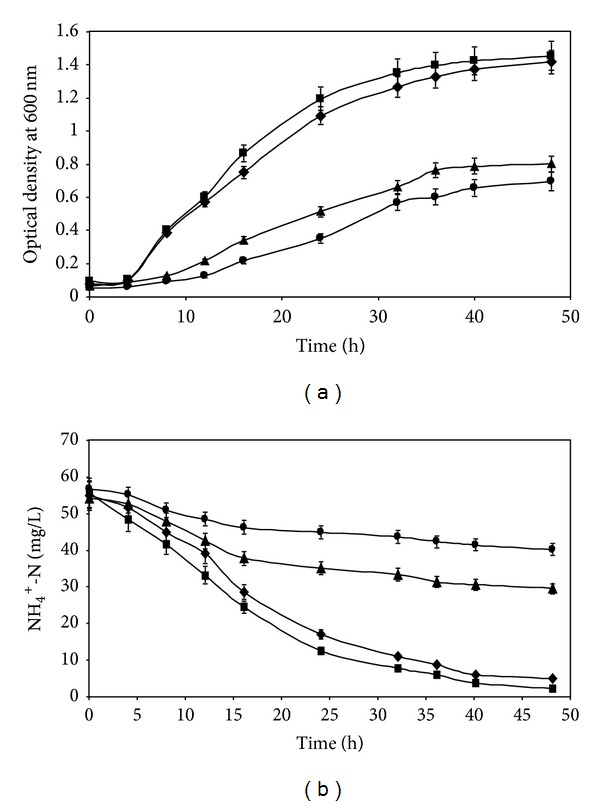
(a) Influence of various carbon substrates on growth of* Chryseobacterium *sp. R31. Sodium succinate* (filled diamonds)*, glucose* (filled squares)*, trisodium citrate* (filled triangles)*, and sodium acetate* (filled circles)*. Error bars represent one SD (*n* = 9). (b) Influence of various carbon substrates on ammonium removal by* Chryseobacterium *sp. R31. Sodium succinate* (filled diamonds)*, glucose* (filled squares)*, trisodium citrate (filled triangles), and sodium acetate* (filled circles)*. Error bars represent one SD (*n* = 9).

The influence of various carbon substrates on growth of strain R31 and ammonium stabilization by the isolate is represented in Figures 4(a) and 4(b), respectively. The plot depicts abundant growth when glucose or succinate was used; whereas lesser biomass was formed with citrate and acetate as substrates indicating that these carbon sources were not utilizable by the isolate. Within 48 h, 95.87% and 91.07% of NH_4_^+^-N were removed in the presence of glucose and sodium succinate, respectively, whilst reduced nitrification occurred in the presence of citrate and acetate. It was also inferred that simultaneous COD removal, nitrification, and denitrification would not be totally achieved in wastewater containing these substrates. Glucose and sodium succinate were superior carbon substrates for nitrogen removal by* Marinobacter *sp. [7] and* Bacillus methylotrophicus* [14] whilst citrate and acetate were inferior substrates, analogous to our results. On the other hand, citrate and acetate proved to be better substrates for nitrogen removal by* A. faecalis* [11]. The four substrates glucose, acetate, citrate, and succinate removed nitrogen with equal efficiency during simultaneous nitrification-denitrification by* Bacillus subtilis* [13].

A specific membrane transporter, citrate lyase activity, and oxaloacetate decarboxylase activity are required for bacterial citrate utilization [38]. There are two routes for the metabolic interconversion of acetate and acetyl-CoA: acetyl-CoA synthetase pathway and acetate kinase-phosphotransacetylase pathway. Growth on acetate further entails the glyoxylate shunt, thus bypassing the decarboxylation steps of the tricarboxylic acid cycle. This, in turn, needs two enzymes, isocitrate lyase and malate synthase [39]. It appeared that the metabolic pathways and/or enzymes required for citrate and acetate utilization were either partially inactive or repressed when isolate R31 was fed with citrate and acetate. Succinate was used as efficiently as glucose for growth and ammonium stabilization by isolate R31. Succinate is a bifunctional substrate of succinate dehydrogenase, an enzyme that is required in both the TCA cycle and the electron transport chain, coupling carbon flow to ATP synthesis [40].

#### 3.2.3. Influence of Nitrogen Substrates on Nitrogen Removal by Strain R31

**Figure 5:**
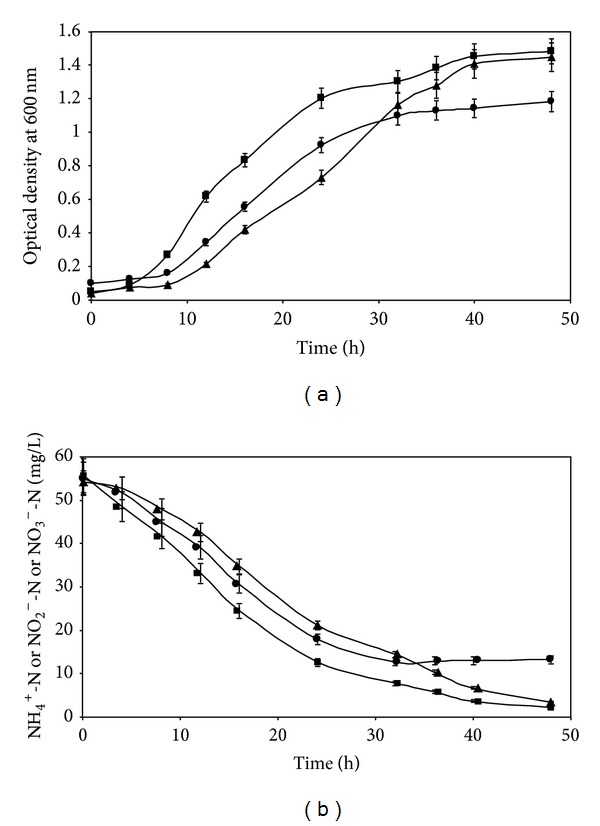
(a) Influence of various nitrogen substrates on growth of* Chryseobacterium *sp. R31. Ammonium chloride* (filled squares)*, sodium nitrate* (filled triangles)*, and sodium nitrite* (filled circles)*. Error bars represent one SD (*n* = 9). (b) Influence of various nitrogen substrates on nitrogen removal by* Chryseobacterium *sp. R31. Ammonium chloride* (filled squares)*, sodium nitrate* (filled triangles)*, and sodium nitrite* (filled circles)*. Error bars represent one SD (*n* = 9).

The influence of various nitrogen substrates on growth of isolate R31 and nitrogen removal is shown in Figures 5(a) and 5(b), respectively. It is evident from the figures that similar growth was attained with ammonium chloride, nitrate, and nitrite as substrates. Nitrogen removal was nearly alike for all three substrates. Thus, the isolate was capable of utilizing ammonium, nitrate, and nitrite as nitrogen substrates and heterotrophic nitrification-aerobic denitrification was independent of the nature of the nitrogen substrate. Growth with nitrite was sufficient, and 75% nitrite was utilized. It appeared that the enzymes required for ammonium utilization such as ammonium monooxygenase and hydroxylamine oxidoreductase as well as nitrite/nitrate reductase required for nitrate utilization were all active when the organism was fed with these substrates. This experiment further confirmed the nitrification and denitrification ability of the isolate. Aerobic denitrification occurring during ammonium removal by R31 demonstrated by utilization of both nitrate and nitrite was similar to the process occurring in* Agrobacterium* strain LAD9 as reported by Chen and Ni [9]. The nitrate removal rate was 1.0 mg/L/h which is higher than that recorded for* Rhodococcus *(0.93 mg/L/h) as reported by Chen et al. [10]. Aerobic denitrifiers utilize nitrite by intracellular assimilation or extracellular reduction pathways. Similar to the observations for* Klebsiella pneumoniae* [16], concomitant cell density increase was noted in our experiments along with consumption of nitrite indicating that extracellular reduction had occurred. Aerobic denitrification ability of strain R31 was confirmed by the positive results obtained in the nitrate and nitrite reductase assays (these enzyme activities were absent in the type of strain of* C. haifense*).

Two pathways are implicated in the simultaneous nitrification and denitrification processes, the first through nitrite and nitrate and the second via hydroxylamine [41].* Thiosphaera pantotropha *[32],* Bacillus subtilis* [13],* Agrobacterium* sp. [9],* Rhodococcus* sp. [10],* K. pneumoniae *[16], and* Marinobacter* sp. [7] possess a complete nitrification and denitrification pathway (NH_4_^+^–NH_2_OH–NO_2_^−^–NO_3_^−^–N_2_O–N_2_). Contrastingly, neither nitrite nor nitrate was detected as intermediates in* Alcaligenes faecalis* [11] and* Acinetobacter calcoaceticus* [15]. Furthermore, nitrite or nitrate reductase activities were not detected in these two bacteria. Denitrification in* A. faecalis* and* A. calcoaceticus* occurs through hydroxylamine, not requiring nitrite or nitrate (NH_4_^+^–NH_2_OH–N_2_O–N_2_). Results as described in this section, in relation to the reports of previous workers, indicate that* Chryseobacterium* sp. R31 has a complete nitrification and denitrification pathway. Conventionally, denitrification occurs under anoxic conditions but strain R31 is obligately aerobic. Microorganisms capable of SND possess respiratory and fermentative types of metabolism. Bacteria belonging to the genera* Agrobacterium* [42],* Rhodococcus* [43],* Alcaligenes* [44],* Acinetobacter* [45], and* Marinobacter* [46] are obligately aerobic. Bacteria of the* Bacillus* genus [47] are aerobic, which may be strict or facultative while the genus* Klebsiella* [48] comprises facultative anaerobic bacteria.

#### 3.2.4. Influence of Different C/N ratios on Growth and Ammonium Removal by Strain R31

**Figure 6:**
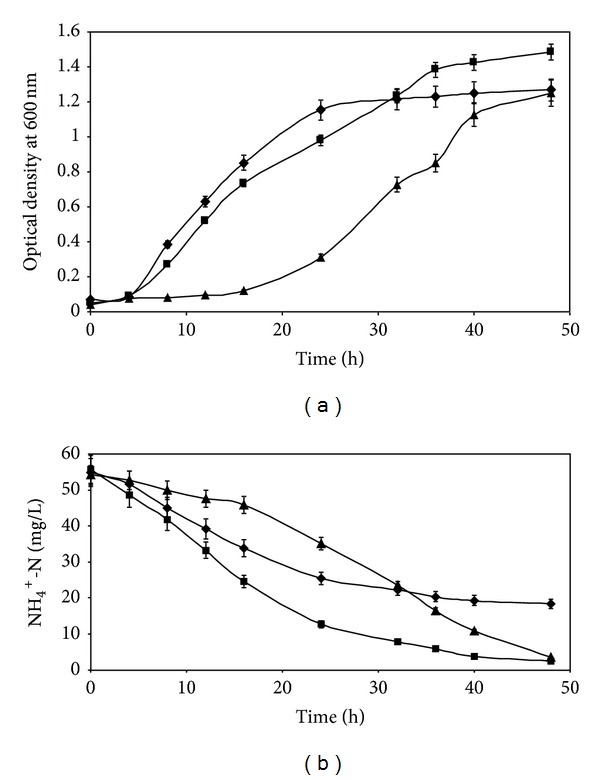
(a) Influence of different C/N ratios on growth of* Chryseobacterium *sp. R31. C/N = 5* (filled diamonds)*, C/N = 10* (filled squares)*, and C/N = 20* (filled triangles)*. Error bars represent one SD (*n* = 9). (b) Influence of different C/N ratios on ammonium removal by* Chryseobacterium *sp. R31. C/N = 5* (filled diamonds)*, C/N = 10* (filled squares)*, and C/N = 20* (filled triangles)*. Error bars represent one SD (*n* = 9).

Figures 6(a) and 6(b) show that, at C/N ratio of 10, the growth rate of isolate R31 was the highest and maximum ammonia nitrogen was removed (95.87%) within 48 h.  Figure 6(b) also demonstrates that, after 48 h of contact, NH_4_^+^-N utilization nearly ceased indicating exhaustion of the nutrient or inhibition of key enzyme(s). The NH_4_^+^-N utilization rate (Figure 6(b)) and growth (Figure 6(a)) of strain R31 were found to be the higher at C/N = 10 in comparison to those achieved with C/N ratios of 5 and 20. The rate of nitrogen removal at C/N = 10 accelerated beyond 8 h and reached an equilibrium level after 40 h. At C/N ratio of 5, the consumption of ammoniacal nitrogen practically stopped after 36 h of incubation, resulting in 63.11% removal. At C/N = 5, heterotrophic nitrification could not be performed efficiently for residual NH_4_^+^-N beyond 20.25 mg/L after 36 h, perhaps due to early exhaustion of the carbon source. On the other hand, at C/N = 10, the growth curve shows that higher biomass was reached probably due to enhanced amounts of utilizable C and N nutrients. At C/N = 20, the overall removal of NH_4_^+^-N was close to the value attained at C/N = 10 but initial consumption was delayed by a time lag. Appreciable removal at this ratio ensued after 24 h. Joo et al. [11] reported similar trend of ammoniacal nitrogen utilization by the heterotrophic nitrifier (*Alcaligenes faecalis* No. 4). Authors observed that, at lower C/N ratio such as 5, 40% unconsumed NH_4_^+^-N remained in the reactor due to dearth of the carbonaceous substrate. At C/N ratio of 10, authors noted that both carbon and NH_4_^+^-N levels decreased in a well-balanced fashion with respect to time along with appreciable amount of growth of the microorganism. For* Bacillus subtilis* A1, optimal C/N ratio was 6.0 and ammonium and acetate were consumed at a proportionate rate [13]. At C/N = 2, the consumption of NH_4_^+^-N stopped because the carbon source was depleted. The NH_4_^+^-N removal efficiency was higher at C/N = 6 and C/N = 12 than at C/N = 2 and C/N = 26 at 60 h.

The optimal values of C/N ratios were 8.0-9.0 for* Agrobacterium* [9], and 15.0 for* Bacillus* sp. LY [10] and* Marinobacter* sp. [7]. Increasing the C/N ratio enhanced nitrate removal rates of* Pseudomonas putida*, whereas nitrogen assimilation into the cell mass was not changed. The optimal C/N ratio was 8 [33]. C/N ratio did not influence heterotrophic nitrification by* Bacillus methylotrophicus* [14]. In general, for a given C/N range, an enhancement in organic carbon concentration resulted in increased denitrification efficiency of aerobic denitrifiers [7]. Our observations support this statement. Lower value of C/N ratio is advantageous in wastewater treatment processes as operating costs can be reduced [9]. Considering this, nitrogen removal using* Chryseobacterium *sp. R31 may be more economical than* Bacillus* sp. LY and* Marinobacter* sp. and as cost-effective as* A. faecalis*. In light of the recommendations of Joo et al. [11], the C/N ratio can be used as a criterion of control when considering ammonium removal in continuous culture by R31 and the addition of external carbon would be necessary in the treatment of high-strength ammonium wastewater at C/N less than 10.

#### 3.2.5. Substrate Removal and Growth Kinetics of Simultaneous Carbon Oxidation, Nitrification, and Denitrification by Strain R31

**Figure 7:**
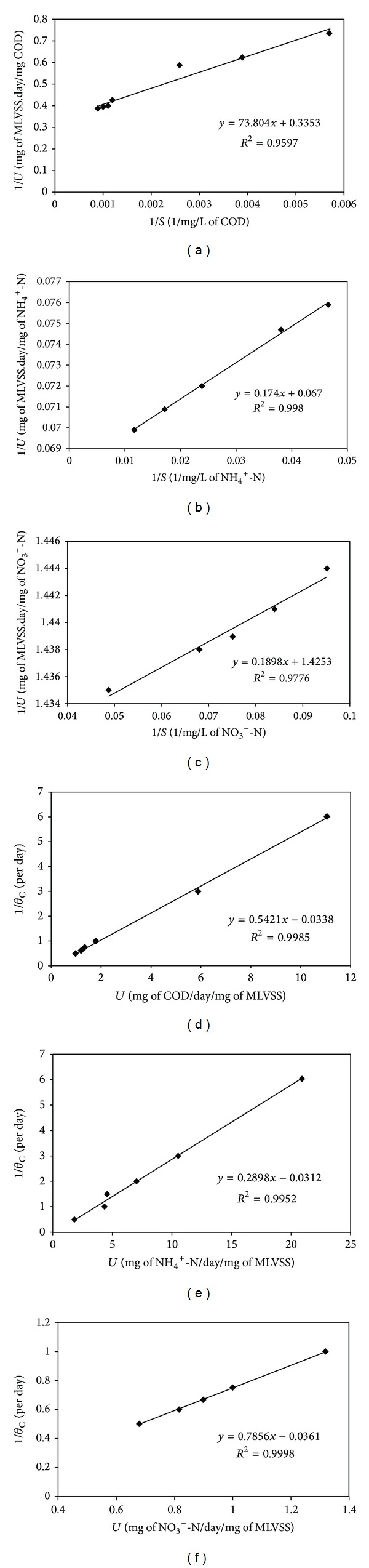
Lineweaver-Burk plot for determination of substrate utilization kinetic constants for (a) carbon oxidation, (b) nitrification, and (c) denitrification and growth kinetic constants for (d) carbon oxidation, (e) nitrification, and (f) denitrification by* Chryseobacterium *sp. R31. Each data point represents the mean value of nine determinations. Error is within one SD of the mean.

Substrate removal kinetics for carbon oxidation, nitrification, and denitrification as well as growth kinetics for carbon oxidation, nitrification, and denitrification were studied applying Lawrence and McCarty's modified Monod equation [34]. The data fitted reasonably well following the Lineweaver-Burk equation (Figures 7(a)–7(f)). From these plots, *K*_*s*_, *k*, *Y*, and *k*_*d*_ were estimated as enumerated in Table 1. As shown in Table 1, the values of the coefficients were similar to those obtained for the other slaughterhouse wastewater isolate,* A. xylosoxidans* [17], and those determined for aerobic-anoxic process for slaughterhouse wastewater [1]. Although belonging to different genera, the kinetics of substrate utilization displayed by the two isolates (*A. xylosoxidans* and* Chryseobacterium *sp.) was essentially similar. It may be noted that the *K*_*s*_ values for carbon oxidation (220.11 mg/L) and nitrification (2.19 mg/L) obtained in this study were higher than those conventionally accepted for domestic wastewater (for comparison see Table 1). Enhanced values were attained due to the higher initial COD and NH_4_^+^-N levels found in slaughterhouse wastewater compared to that present in domestic wastewater [18]. Values indicate that R31 had low affinity for carbonaceous substrate as well as for ammonia. The high *K*_*s*_ value may be an effect of high substrate concentration [49]. The other kinetic coefficients (*k*, *Y*, and *k*_*d*_) for carbon oxidation and nitrification were, however, comparable to that reported for domestic wastewater [18]. For municipal and industrial wastewater, the coefficient values represent the net effect of microbial kinetics on the simultaneous degradation of a variety of different wastewater constituents. The kinetic coefficients determined for denitrification corroborated with the typical values obtained for domestic wastewater (for comparison see Table 1). The constants determined are suitable for COD and nitrogen removal, similar to the conclusions of Pala and Bölükbaş [50]. Authors reported that the Monod kinetic constants (*Y*, *k*_*d*_, and *K*_*s*_) were indicative of efficient COD and nitrogen removal from municipal wastewater. The kinetic coefficients obtained in this study are supportive of those calculated by Kundu et al. [1]. This proves that R31 has similar capacity to perform in terms of COD and nitrogen removal from slaughterhouse wastewater if used in a large-scale SND treatment plant. Kinetic coefficients for simultaneous carbon oxidation, nitrification, and denitrification by any* Chryseobacterium *species are being determined for the first time. Analysis of the kinetic coefficients is the best method for prediction of potential large-scale application of pure cultures and this approach has been described for the first time for bacteria capable of SND.

The* Chryseobacterium* genus was first described by Vandamme et al. [51] with six species and, following the revised description of the family Flavobacteriaceae by Bernardet et al. [52], more than sixty novel species of the genus* Chryseobacterium* have been identified from a large number of sources such as wastewater [53], contaminated soils [54], plant rhizosphere [55], human clinical source [56], chicken [57], fish [58], mosquito [59], glacier [60], and beer manufacturing [61]. However, most of the novel species have been reported from bovine milk [36, 62, 63]. Our isolate was obtained from slaughterhouse wastewater for the first time, and, to the best of our knowledge, nitrification and denitrification property within this genus is also being recorded for the first time. The* Nitrosomonas* genus having ammonia-oxidizing bacteria and the* Nitrobacter* genus comprising nitrite oxidizing bacteria are regarded as the principal genera of chemolithotrophic nitrifying bacteria. Accordingly, the probes required for fluorescence in situ hybridization (FISH), NSO1225, and NIT3 were selected to stain *β*-*proteobacteria *sp. AOB and* Nitrobacter *sp. NOB, respectively by Pan et al. [6] during their study on nitrogen removal from abattoir wastewater through partial nitrification and subsequent denitrification in intermittently aerated sequencing batch reactors. Authors justified the selection stating that both *β*-*proteobacteria *sp. AOB and* Nitrobacter *sp. NOB were widely found in wastewater systems. However, Yilmaz et al. [8] noted that common NOBs (*Nitrobacter* and* Nitrospira*) were not found in the sequential batch reactor during simultaneous nitrification, denitrification, and phosphate removal of abattoir wastewater. In consonance with the finding of Yilmaz et al. [8] and similar to our previous study [17],* Nitrosomonas*,* Nitrobacter*, and* Nitrospira* were not recovered as the most active nitrifiers/denitrifiers in the current investigation. Our two studies assert that novel microorganisms should be considered for simultaneous COD reduction, nitrification, and denitrification in slaughterhouse wastewater.

## 4. Conclusions

Through the present investigation a bacterium isolated from slaughterhouse wastewater was identified as* Chryseobacterium* sp. which successfully stabilized COD as well as ammonium nitrogen. The bacterium was capable of utilizing NO_3_^−^-N aerobically in presence of NH_4_^+^-N. Nitrogen removal was dependent on the nature of the carbon substrate but independent of the type of the nitrogen substrate. This bacterium is a new member in the group of microbes capable of simultaneous removal of organic carbon and nitrogen from wastewater. Further taxonomic investigations are warranted to establish the strain as a new species. The kinetic coefficients of substrate removal and growth for concomitant carbon oxidation, nitrification, and denitrification are favorable and corroborative with the results of earlier researchers. The kinetic coefficients may be considered for the design of bioreactors, thus opening up the possibility of SND process in treatment of slaughterhouse wastewater.

## Supplementary Material

Supplementary Material 1 shows the nitrification activities of all ten isolates and taxonomic affiliation of some isolates. Supplementary Material 2 represents the morphological and physiological characteristics of Chryseobacterium sp. R31. Supplementary Material 3 shows the results of the identity analysis of strain Chryseobacterium sp. R31 based on 16S rRNA gene sequence performed on the EzTaxon server (last accessed on 20/02/2014). 1373 bp 16S rRNA gene of Chryseobacterium sp. R31 has NCBI Accession No. KF751764. Supplementary Material 4 describes the long chain fatty acid composition of the isolate R31.

## Figures and Tables

**Table 1:** Summarized values of kinetic coefficients obtained in the present study, our previous study [17], and domestic wastewater [18].

Kinetic coefficients	Carbon oxidation			Nitrification			Denitrification	
	Present study	Kundu et al. [17]	Metcalf and Eddy [18]	Present study	Kundu et al. [17]	Metcalf and Eddy [18]	Present study	Metcalf and Eddy [18]
*k* (day^−1^)	2.98	2.24	2–10	14.66	13.66	1–30	0.70	0.23–2.88
*K*_*s*_ (mg/L)	220.11	232.0	25–100	2.19	2.13	0.2–0.5	0.13	0.06–0.20
*Y* (mg/mg)	0.54	0.44	0.4–0.8	0.28	0.24	0.1–0.3	0.78	0.4–0.9
*k*_*d*_ (day^−1^)	0.03	0.06	0.025–0.075	0.03	0.05	0.03–0.06	0.03	0.04–0.08
