# Supplementary material for: Simultaneous Heterotrophic Nitrification and Aerobic Denitrification by Chryseobacterium sp. R31 Isolated from Abattoir Wastewater
Source: Biomed Res Int. 2014 Jun 2;2014:436056. doi: 10.1155/2014/436056 (PMC4060765; doi:10.1155/2014/436056)
Supplement: Supplementary file 1 — Supplementary Material 1 shows the nitrification activities of all ten isolates and taxonomic affiliation of some isolates. Supplementary Material 2 represents the morphological and physiological characteristics of Chryseobacterium sp. R31. Supplementary Material 3 shows the results of the identity analysis of strain Chryseobacterium sp. R31 based on 16S rRNA gene sequence performed on the EzTaxon server (last accessed on 20/02/2014). 1373 bp 16S rRNA gene of Chryseobacterium sp. R31 has NCBI Accession No. KF751764. Supplementary Material 4 describes the long chain fatty acid composition of the isolate R31. [file 436056.f1.pdf]

Supplemenatry File 1

**Isolate No.**	**Colony size**	**Colony pigmentation**	**Colony form**	**Colony margin**	**Colony elevation**	**Taxonomic affiliation based on 16S rDNA sequence**	**Ammonium nitrogen removal (%)**
R11	Large	White	Irregular	Serrate	Flat	In progress	48.57
R12	Moderate	Light Yellow	Circular	Entire	Flat	*Bacillus* sp.	51.24
R13	Moderate	White	Circular	Entire	Umbonate	In progress	55.32
R14	Small	White	Circular	Entire	Raised	In progress	52.41
R15	Large	White	Irregular	Undulate	Flat	In progress	67.54
R16	Small	White	Irregular	Entire	Raised	In progress	58.22
R30	Moderate	Light Yellow	Circular	Entire	Flat	In progress	64.21
R31	Moderate	Yellow	Circular	Entire	Flat	*Chryseobacterium* sp.	95.87
R32	Moderate	White	Circular	Entire	Flat	*Exiguobacterium aurantiacum*	61.25
S18	Pin Point	White	Circular	Entire	Raised	*Achromobacter xylosoxidans*	97.2

Supplementary file 2

Morphological and physiological characteristics of *Chryseobacterium* sp. R31

Morphology

Colony morphology (nutrient agar)	Circular, yellowish, opaque, entire edges
Motility	Non-motile
Gram-staining	Negative

Growth conditions

Temperature range	Good growth at 25–42 ºC
pH range	6.0–7.5
Relation to oxygen	Obligate aerobic
Growth in the presence of 7% NaCl	Positive

Physiological characteristics

Negative	Acid production from sucrose, lactose, xylose, sorbitol, inositol, trehalose and manitol, catalase, urease, lysine decarboxylase, *o*-nitrophenyl-β-galactoside, DNA hydrolysis, Indole test, flexirubin pigment, starch hydrolysis, Voges Proskauer test, methyl-red test, Simmons citrate
Positive	Acid production from glucose, fructose, arabinose, maltose, galactose and mannose, indophenols oxidase, nitrate reduction, arginine dihydrolase, aesculin hydrolysis, gelatin liquefaction, Tween 80 hydrolysis, tyrosine hydrolysis, H_2S production

Sole carbon source utilization test

Negative	Sucrose, lactose, xylose, sorbitol, inositol, trehalose, mannitol, phenol, ethanol, sodium citrate and sodium acetate
Positive	Glucose, fructose, arabinose, maltose, galactose, mannose, glycerol, methanol and sodium succinate.

Sole nitrogen source utilization test

Negative	Urea, cobaltous nitrate and casamino acid
Positive	Ammonium molybdate, ammonium nitrate, calcium nitrate, potassium nitrate, ammonium chloride, ammonium sulphate, sodium nitrate, sodium glutamate and peptone

Amino acid utilization test

Negative	Methionine, glysine, aspartic acid, glutamic acid, glutamine, asparagine, leucine and cystine
Positive	Tyrosine, tryptophan, serine, isoleucine, OH-proline, lysine, histidine, arginine, cysteine, valine, phenylalanine, threonine, proline and alanine

Antibiotic susceptibility/resistance characteristics

Susceptible	Carbenicillin, oleandomycin, chloramphenicol, tetracycline, kanamycin, lincomycin and vancomycin
Resistant	Gentamycin, streptomycin, ampicillin, penicillin-G and neomycin

Supplementary File 3

Results of the identity analysis of strain *Chryseobacterium* sp. R31 based on 16S rRNA gene sequence performed on the EzTaxon server (last accessed on 20/02/2014). 1373 bp 16S rRNA gene of *Chryseobacterium* sp. R31 has NCBI Accession No. KF751764

Rank according to similarity	Species Name	NCBI Accession No.	Pairwise similarity (%)	Dissimilar nucleotides/total nucleotides
1	*Chryseobacterium haifense* H38^T	EF204450	98.25	24/1371
2	*Amoebinatus massiliae* 7Ebis	AF531765	97.60	33/1373
3	*Chryseobacterium anthropi* NF 1366^T	AM982786	97.52	34/1369
4	*Chryseobacterium pallidum* 26-3St2b^T	AM232809	96.72	45/1371
5	*Chryseobacterium carnis* NCTC 13525^T	JX100817	96.70	45/1365
6	*Chryseobacterium hominis* NF802^T	AM261868	96.43	49/1372
7	*Chryseobacterium molle* DW3^T	AJ534853	96.42	49/1369
8	*Chryseobacterium hispanicum* VP48^T	AM159183	96.34	50/1366
9	*Chryseobacterium koreense* Chj707^T	AF344179	96.06	54/1371
10	*Chryseobacterium gambrini* 5-1St1a^T	AM232810	95.81	57/1360
11	*Chryseobacterium treverense* IMMIB L-1519^T	FN297836	95.70	59/1373
12	*Chryseobacterium piscicola* VQ-6316s^T	EU869190	95.63	60/1373

Supplementary File 4

Long chain fatty acid composition of the isolate R31

Sl. No.	Fatty Acid	Percent (%)
1	13:0 iso c	4.22
2	13:0 anteiso c	0.39
3	14:0 iso c	0.86
4	15:0 iso c	40.40
5	15:0 anteiso c	19.44
6	16:0 iso c	1.04
7	Sum In Feature 3	4.10
8	15:0 iso c 3OH	3.12
9	15:0 c 2OH	1.09
10	17:1 iso c ω9c	9.94
11	16:0 iso c 3OH	1.53
12	18:1 iso c ω5c	0.65
13	17:0 iso c 3OH	11.13
14	17:0 c 2OH	2.07
